# Competing discourses as barriers to change in rehabilitation nursing: a discourse analysis

**DOI:** 10.3389/fresc.2023.1267401

**Published:** 2023-12-12

**Authors:** Sanne Angel, Randi Steensgaard, Raymond Kolbaek, Søren Frimann

**Affiliations:** ^1^ Research Unit of Nursing and Healthcare, Department of Public Health, Faculty of Health, Aarhus University, Aarhus, Denmark; ^2^ Specialized Hospital for Polio and Accident Victims, Aarhus, Denmark; ^3^ Department of Culture and Learning, Aalborg University, Aalborg, Denmark

**Keywords:** action research, biopsychosocial, discourse analysis, nursing, organization, patient participation, rehabilitation

## Abstract

**Introduction:**

The power of action research to create change by anchoring research results in practice was challenged in an action research project at a specialized rehabilitation unit for persons with acquired spinal cord injury. Despite the co-researchers' new insights, approaches, and actions supporting patient participation, it was not possible to change the basic conditions for the practicing of nursing. We aimed to raise awareness of the mechanisms that govern barriers by exploring these barriers as experienced by nurses in their effort to change their practice to improve patient participation.

**Method:**

We used Fairclough's critical discourse analysis drawing on Foucault's practical systems; ethics (identity, relation to oneself), power (action, relation to others), and knowledge (representation, aspects of the world), which he combines with discourse-analytical concepts.

**Results:**

Our discourse analysis of the empirical data at micro-level uncovers the nature of barriers to change in practice. In addition, our analysis at macro-level unveils how these practices are embedded in larger historical, societal, and institutional discourses. This identified two current discourses: a biomedical discourse and a biopsychosocial discourse. In the light of these two discourses, the nurses at micro-level saw themselves as strong agents for the best rehabilitation by acting in accordance with the biopsychosocial discourse. But they were unable to find the time and space to do so due to tasks, structures, and practices specified by an organization dominated by the biomedical discourse.

## Introduction

This article explores challenges in changes resulting from an action research (AR) study investigating nurses' possibilities to facilitate patient participation in a Danish rehabilitation unit for patients with spinal cord injury (1, 2). Despite the co-researchers' new insights, approaches, and actions supporting patient participation, it was not possible to change the basic conditions for nursing practice. We problematize this because the AR study emphasized the importance of previous evidence that patient participation in rehabilitation nursing supports patients' possibilities to continue a meaningful life after spinal cord injury as also reported by Angel (3). Life after spinal cord injury often requires adjustments and a changed reality regarding family, social, and work life as well as the ability to gain independence (4). This adjustment benefits from the injured patient's engagement in rehabilitation because successful outcome depends on the patient's participation (5–7). Therefore, participation is crucially important for the patient's process of achieving a life worth living (8–10).

The importance of patient participation in rehabilitation is recognized in nursing. The close connection between patient participation and care is centered around the patient's perspective as outlined in several studies (7, 11–13). This approach has been conceptualized as patient-centered care (14), and aims at enhancing the person's autonomy, self-determination, independence, empowerment, and health (11, 7, 14–17). On this basis and the recognition of the tendency of not meeting patients' needs for fundamental care, Kitson et al. (18) developed a framework for person-centered care. They argued that care is under pressure due to a mechanistic and depersonalized approach to the basics of care, termed as “task and time driven culture” (12, 16, 18). Kitson et al. (16) conceptualized their thoughts in the conceptual framework “Fundamentals of Care”, which was also presented as a theoretical frame for nursing at the spinal cord injury center in this study?

During the AR study by Steensgaard (1), the involved nurse co-researchers were surprised how little they knew about the patient's situation, perspective, and preferences and how little this knowledge had been included in their nursing so far (1, 2). The AR study showed that the patient's perspective was of major importance to the nurses to adjust their care to the patient's needs. This cognition made co-researchers achieve a deeper commitment to the rehabilitation of each patient. This engagement made the co-researchers realize how their values as professional nurses had been suppressed by organizational structures in their daily work. Because of the AR process, the co-researchers rediscovered the core of their profession and became enthusiastic about incorporating the patient's perspective to enhance care and support the patient’s return to a meaningful everyday life.

The challenges in changing the nurses’ practice were even more surprising because their professional ambition of caring for the patient's whole existence and meaningful life was similar to the goal of rehabilitation (20, 21). Yet, they were prevented from pursuing this approach although the specialized rehabilitation unit was expected to have a biopsychosocial approach as described in the International Classification of Functioning (ICF) model. This approach takes both biomedical, psychological, and social aspects into account when identifying patients' rehabilitation needs (22). Thus, the nurses' expectations to their contribution to rehabilitation pointed to a meaningful life (22) and aimed at “assisting individuals, who experience or are likely to experience disability, to achieve and maintain optimum functioning in interaction with their environments” (23). The WHO definition says “rehabilitation addresses the impact of a health condition on a person's everyday life, by optimizing their functioning and reducing their experience of disability” (20). To reduce the negative consequences of a spinal cord injury, the task of highly specialized rehabilitation units is to provide tailored rehabilitation based on relevant professionals' assessments of the patient's potential for rehabilitation. This underlines the importance of engaging the patient. Thus, nurses' experiences of difficulties in engaging patients in their care called for a closer look into the organization of a highly specialized rehabilitation setting.

The need to engage the patient was highlighted by Kirkevold (23) and described as nurses' therapeutic function; (1) Interpretive; elaboration of what had happened and what that means to the patient, (2) Consoling; emotional support related to distress and loss in the difficult situation, (3) Conserving; protecting physical integrity, function and preventing complications, (4) Integrating; helping patients integrate newly learned activities to gain a well-functioning everyday life. These therapeutic roles and functions are consistent in rehabilitation nursing (24). A dialogic approach to the patient is common in all of these functions. This was also underlined by the result of our AR study showing the need to prioritize dialogue with the patient to promote the patient's participation (1, 2). Within the AR design, we experimented with how to make the needed changes to provide time and space for the necessary dialogue. The study was based on the power of involving health professionals as this would positively affect barriers to implementation and changes in practice (25–27). However, the dialogue was only possible when the nurses succeeded in creating extraordinary space between mandatory nursing tasks. We searched the data again to understand the barriers to changing this practice to support patient participation.

## Aim

To gain awareness of the mechanisms governing the barriers of change by exploring how these were experienced by nurses in their effort to change their practice to improve patient participation.

## Methodology

This discourse analysis study is a follow-up on a larger AR project investigating how nurses can contribute to patients' participation in rehabilitation.

### The action research study

The project and its processes used various methods, such as observations, meeting minutes, and written logs of nurses' reflections on their work. These reflections were discussed in workshops and consecutive meetings. The data from these sources formed the basis for actions in practice.

The AR study was carried out in line with the dialogue tradition in AR, which has primarily been developed in the Scandinavian countries (28–32). This tradition has a process-oriented perspective experimenting with participation, organizational learning, and empowerment through dialogues. The primary methods are called *dialogue conferences* and *the development organization* allowing everybody in an organization to participate in processes of development, improvement, and innovation*.* Members of the organization step out of their usual roles to critically evaluate the organizational practices in a dialogically reflective space, where they focus on investigating, clarifying, developing, and learning at individual, team, and organization level (33). The strength of this approach is the focus on developing organizations through actions in practice, thus creating local theories and “actionable knowledge” (34). The novelty of this dialogue tradition is that changes in practice through AR projects are seen as a result of using words in dialogues and communication, which create new forms of local discourses and language, resulting in a re-organization of discourses and practices (35).

### Setting

The study took place at a Danish highly specialized rehabilitation center for patients with a spinal cord injury. The center is related to a hospital. Approximately 100 patients are admitted annually and the center has 35 beds. The center employs more than 100 inter-professional staff members, including doctors, secretaries, psychologists, social workers, occupational therapists, physiotherapists, nurses, and nursing assistants.

An advisory board consisting of a representative from the co-researchers, one former patient, the supervisors, and representatives from the nursing and inter-professional managers facilitated the process and anchored the project.

### Data collection

Data for the discourse analysis was selected among the AR data collected in the period between 2016 and 2018. The AR data consisted of the log-book notes of eight nurse co-researchers, four one-day workshops, and nineteen one-hour meetings. All meetings and workshops were audio-recorded and transcribed verbatim.

For the discourse analysis, we selected passages where the nurses were challenged and thus problematized their effort to engage the patient to participate. This data material consisted of 66 transcribed pages for analysis and discussion among the authors. The data material was reviewed, categorized and coded in relation to semantic choices, themes and discourses realized through the nurses' use of language.

### The follow-up discourse analysis study

In accordance with both Foucault (36) and Fairclough (37), consistently discursive patterns can be found in qualitative data, as they are constructed in the form of identities, positions, values, perspectives, representations of knowledge, power relations, conflicts, and barriers. These discourses often also have a history, such as the archaeological perspective of Foucault (36), and the discourses may also express an organizational culture and power relations. From a macro-perspective, discourses can be related to the larger institutional and societal discourses such as the New Public Management discourse, the co-creation discourse, the patient-centered discourse, etc. In the broader field of discourse analysis with many different approaches (38), Fairclough (39) divides the analytical approaches into a micro- and a macro-oriented approach.

The micro-field includes text-oriented approaches to discourse analysis (TODA), which perform close discourse analysis at micro-level such as conversation analysis, multimodal analysis, social semiotics, discourse-oriented ethnography, and narrative analysis. This allows us to closely analyze the specific linguistically construed discourses in our data material at micro-level such as the construction of themes or discourses, positions, power, identities, and relationships between nurses, patients, management, and other professional groups created as a result of observations and nurses' reflections. Analysis of the social practice at macro-level allowed us to analyze the orders of discourse such as the larger institutional, scientific, societal, and historical discourses in which the concrete practices are embedded. The macro-field constitutes social-oriented approaches to discourse analysis, (SODA), which focus on the social and historical aspects of discourse. Fairclough (40) defines discourse in an abstract societal sense as *Discourse (capital D)* and in a concrete sense, *discourse* (lower case d). He defines *Discourse* as “language used as a social practice”, while *discourse* is defined as a specific way of linguistically “representing aspects of the world” (40). Discourse approaches assume that language use is a constitutive part of any social practice. We construct our social world by the way we use language and through the meanings we ascribe to situations, activities, and larger organizational, political, or ideological events. “*Outside discourse there is no social reality, and if we cannot understand discourse, we cannot understand our reality, our experiences, and ourselves*” (41).

### Analysis

Starting with the micro-oriented approach, we chose to search for everything describing barriers to change using critical discourse analysis. We analyzed the empirical data to discover the nature of barriers to change in practice. At the same time, these concrete practices are also embedded in larger historical, societal, and institutional discourses, which can create barriers and be more difficult to change as time and effort are needed to change organizational and cultural barriers. We thus examined the relationship with the specific practice and looked for a deeper understanding of what could be changed within the organizational framework (macro-oriented approach). To illustrate the challenges, we present a case based on observations of a typical real-life scenario.

### Case based on a real-life scenario

A nurse enters a patient's room at 7.30 am. The patient has been awake for a while because he used to get up at 6 to be ready for work—before the injury. Now his body is stiff and hurts, and he cannot wait to get out of bed.

The nurse enters the room with energy and tries to spread a good and cozy atmosphere by small talking, smiling, and joking. A plan for the morning is presented to the patient; “besides you, I also have another patient and I have to prepare for rounds”.

The patient says that he must be ready for training at 9.30 and that he has been late several times this week.

The nurse avoids commenting and immediately starts preparing for emptying the bladder with a catheter, preparing a commode, and finding towels, and clothes. While she is lifting the patient, her phone rings. The nurse stops the procedure to pick up the phone with the patient sitting naked on the commode with a small towel to cover his private parts.

The patient asks to take a shower while emptying the bowel to save time. The nurse stays in the bathroom because she needs to assist the patient with bathing. At this point, neither the nurse nor the patient can leave the room, and the nurse takes the opportunity to ask the patient about his life situation; how everything works at home, and how the plan for selling the house is progressing (a necessity because the patient is too disabled to get back to his former house) and how the patient feels about going home for the weekend.

When the patient is back in bed ready to get help with the clothes, the nurse's phone rings again. The nurse leaves the room after a short conversation over the phone telling the patient that she will return as soon as possible.

The patient asks her not to take too long because time is running, and he will almost certainly be late. The tone is appealing and on the edge of demanding.

After 20 min the nurse returns. The atmosphere is tense now and the patient comments on her late return. The patient is rushed into his clothes, a quick comb through the hair, tooth brushing—shaving must wait until later or another day. It is 9.25 am. The patient leaves the room in a hurry. Breakfast will have to wait or just be skipped for the day. The nurse rushes to finish off helping her other patient get ready for his training, before doing rounds, attending meetings, and documenting her nursing. The nurse attends to the patient one more time that day to empty the patient's bladder.

We used critical discourse analysis (39, 40) drawing on Foucault's (36) practical systems; ethics (identity, relation to oneself), power (action, relation to others), and knowledge (representation, aspects of the world), which he combines with discourse-analytical concepts. This results in three functions where people use language: a) to construe identity, b) to construe relationships, and c) to represent aspects of the world. This analysis was guided by a discursive thematic analysis framework (36, 38–40). We analyzed how rehabilitation nursing is being displayed? and how it is linguistically constructed using the data from the actual situation and how this collides with the nurses’ understanding of rehabilitation nursing. Therefore, we read and re-read selected data describing barriers to get a sense of the whole. Then we read the text to analyze nurses' language with a focus on the following three functions:

A: **Identity function**. Wordings that point to the speaker's or writer's construction of identity, role, or position at work in the semantic choices (in **bold**) and in linguistic modality markers (adverbs, modal verbs, and modal phrases), which indicate how certain the speaker is of his or her statement (underlined). The following excerpts illustrate the nurses' dialogues and reflections on their understanding of their professional identity and role.

Example 1 (Workshop 1):

CR: (…) *I actually also think it’s a bit about the fact that we **have to sharpen our senses** because I actually often think that patients, once they know us, they **send some signals** that we **must then be able to pick up**: “Hey, I actually just want to talk to you today or I just have something on my mind or..”. And then we **have to go into it at this point?** because I think that’s where they need it*.

Example 2 (Co-researcher meeting 7)

CR: (…) *often I have noticed that I thought I should **come up with solutions** and that I should then **be able to help them out of it?**. When I **have really concentrated**, it is actually often (…) he then just simply had **a huge need to tell me what he was feeling right now and how he was experiencing his life right now**; that I **wanted to listen to him**. So that was what he was asking for*.

Example 3 (co-researcher meeting 10)

A: (…) *part of our finest nursing is caring for our patients. And in the care is the conversation* (…)

In these examples, nurses are in the process of reflecting on a new understanding of their professional role and identity. This includes showing care by being a good dialogue partner, who has time to listen to the patient's feelings and experiences without making suggestions, solutions, or helping to solve the patient's problems. As the new understanding of the professional role is evolving, the co-researchers mark their statements with a lower degree of certainty and thereby an openness to interpersonally negotiate their new identity and role in the dialogues: *think, actually, often, a bit, thought,* etc.

**B: Relational function**. The linguistic construction of relationships, actions, and power in relation to others e.g., interaction patterns such as question-answer, topic control, word distribution, and interruptions. Speech acts such as “suggest”, “order”, “express”, “ask”, “warn”, “joke”, “claim” (in **bold**). Personal pronouns such as “I”, “you”, “one, “they”, and “we” (underlined) are used to construe participants’ relationship and responsibility in the communication.

Example 4 (Co-researcher meeting 16)

*(…) One also thinks that if it's [dialogue with the patient] important that it's part of the rehabilitation process here at the rehabilitation unit, that it is part of the package, one can also **turn it around** and say: Why is it that we have to **put so much effort into trying to (…) get it rescheduled?** [agreements and appointments]? **Instead of being able to say to the patient**: Today, you are **not going to your training**, because you and I **are going to have a dialogue***.

A: *Simply squeeze it in [to schedule tasks]. Sometimes one can also turn it around completely and say (…) why is it not just as valid to say: “we have a conversation today”*.

Example 5 (co-researcher meeting 7)

*(…) **just time to be**. That it's not always at the same time as all kinds of practical things (…) It has **always been** our **big problem** that we should just **do something at the same time** [as having a dialogue]. So, it's also **a challenge to just sit down and talk sometimes**, because we may have **10 other tasks waiting**, and then we also **have to find a calm moment? to go in and have a dialogue with the patient***.

These examples construe the relation and collaboration between the nurse and the patient and the other professions in the unit. Nurses find it difficult to find space and room for dialogue with the patient among other tasks. The extracts consist of speech acts e.g., *squeeze it in, turn it around, have a dialogue, make time to just be, sit down and talk,* etc. These suggestions call for changes in the organization of the work and implicitly involve management and power to change the organization of tasks. It also involves incorporating the biopsychosocial discourse in rehabilitation. How the speech acts are anchored in personal pronouns is interesting, as the requests or demands for re-organizing the discourses of rehabilitation are made through the distancing impersonal pronoun “one”. This means that the responsibility of suggested actions is vaguely anchored in the discourse i.e., you do not know exactly *who* is responsible for these speech acts. Expressions of experiences, challenges, and problems in the nurses' attempt to make room for dialogue with their patients are made by using the personal pronouns *I, we,* and *ours*. Thus, the responsibility for utterances and their consequences are clear.

**C. Representational function.** Wordings are used to represent knowledge or aspects of the world in semantic choices i.e., words—especially nouns and verbs (in **bold**), and metaphors (underlined) used to represent content. The following are examples of co-researchers' evaluation of experiments with new ways of working with rehabilitation through dialogues with the patient and by motivating the patient to participate in his or her own rehabilitation.

Example 6 (workshop 1)

CR: *But then it is the “**care time”** we are **dealing with**. So, when you look at it, then this may be the **next experimenting action**. The “**care time” must be documented** [as a work task] **in some way***.

CR: *I'm just thinking we also need **be aware of** (…) I was just about to say [aware of] **their life story, their way of life, their mental state**. So, it also **changes over time while they are with us** [admitted to the* rehabilitation unit*]. So, they also **fluctuate depending on how many resources they have, how strong they are, how motivated they are. There are some phases*****.**

Example 7 (evaluation of experimenting action 2)

CR 1: (…) *the patient gets a picture of the whole situation and reflects on how he/she is doing*.

CR 2: (..) *it allows the patients to articulate their own thoughts, feelings, hopes and concerns. I think it makes a big difference to have said it to someone else, rather than to be alone with it*.

CR 3: *This is the **code for drive, motivation, energy, and problems**. **Doubts and frustration** can **emerge**. [It is important to] **Build a relationship**. Here the pieces of the puzzle are turned and rotated. Maybe a few pieces are put in place, but that is not the goal. The goal must be to get them into play and to locate them [the pieces]*.

In these examples, experimenting actions represent a biopsychosocial discourse in rehabilitation. It is referred to by the word “*care*”, which takes place as dialogues between the nurse and the patient about his/her current situation, motivation, life story, hopes, frustrations, problems, and variations in the rehabilitation process. The biopsychosocial discourse emphasizes creating a dialogic space, a more holistic and complete picture of the patient's situation, and a good relationship between the nurse and the patient; this may be emancipating, create motivation, and be a starting point for the patient's responsibility for involvement and participation in the rehabilitation. The puzzle metaphor is a picture of how to work with patients through dialogue to create a picture of the whole situation. Furthermore, the dialogic “care time” “*must be documented”*; this points to the challenges with the understanding based on a biomedical discourse concerning rehabilitation. The work was organized according to this discourse; here no time was made for dialogues between the nurse and the patient. With the development of the new biopsychosocial understanding, it becomes a requirement to plan with and incorporate time for dialogue with the patient in the nurses' tasks. Implicitly, this is about changing the discourse and understanding of rehabilitation, which must be implemented in the organization of the work and the general scientific discourse on rehabilitation.

From the specific linguistically construed discourses in our data material at micro-level, we found that in the concrete practices, the larger historical, societal, and institutional discourses of rehabilitation were embedded at macro-level. This left us with two simultaneous and competing discourses: a biomedical discourse and a biopsychosocial discourse. In the light of these two discourses, the nurses at micro-level saw themselves as strong agents in providing the best rehabilitation by acting on the biopsychosocial discourse but being unable to find the time and space to do so due to an organization based on the biomedical discourse. This demands a new organizational focus to allocate time, which will involve a change in other professionals' workflow and use of resources.

## Results

The AR study explored how nurses could promote patient participation and showed that it was possible to work with a biopsychosocial understanding to rehabilitation. This also entailed a renewed and enhanced professional identity and role for the nurses in their relational work with the patients.

The patient-nurse dialogue proved to be central for the co-researchers to obtain an in-depth understanding of the patient as an individual as well as to build a trusting and cooperative relationship. Furthermore, the patients recognized their situation through dialogue, which led to motivation and actions in the rehabilitation process. The dialogic space thus led to? understanding, learning, and cognition. The joint dialogue between the nurse and the patient also led to a mutual reconciliation of expectations and adjustment of services, resulting in progress, motivation, and thus a more individualized rehabilitation. Involving the patient's special circumstances proved to be a stronger approach to rehabilitation than the dominant one-size-fits-all biomedical approach.

More dilemmas appeared from the co-researchers' realization that patient participation required time and space for the patient's voice to be heard to adapt nursing interventions and promote the patient's rehabilitation. This was considered of utmost importance in the co-researchers' understanding of rehabilitation to ensure that the patient could obtain a level of physical functioning to live a meaningful life. Unfortunately, this contrasted with existing procedures involving a tight time regime to which both patients and health professionals had to conform. Thus, the focus was on finishing e.g., the morning task-based routines and physical training instead of involving patients in the tasks, which could contribute to mental and physical improvements. Therefore, the need for both time and space for dialogue was particularly important. It should not be “something extra” that nurses had to find time for in addition to their existing tasks, which would lead to increased work pressure. The revealed contrasts between the biomedical and the biopsychosocial discourse led to dilemmas and choices had to be made.

### Finishing on time vs. integrating all activities in rehabilitation

The time pressure to get patients ready for training meant that nurses had developed coping techniques resembling assembly line work to help more patients at the same time. The nurses had to focus on finishing practical tasks, for making it possible for therapists to train with the patients and thus improve their physical condition. This practice is illustrated by the case described above. Nursing aims to improve patients' overall situation, including both biological, psychological, and social well-being, and importantly that patients feel seen and heard and thus acknowledged as valuable human beings.

As illustrated by the case, when the nurse was not in a position where she could meet the patient's psychosocial needs, she was careful not to open a discussion about deeper needs, which could not be managed within the time frame while preparing the patient for physiotherapy. Due to time constraints, the potential of using the morning routine to improve the patient's physical capacity was not exploited. Furthermore, the possibility to expand the interpersonal relations between the nurse and the patient could have positively promoted the patient's experience of being acknowledged as a human being and not just as a part of an assembly line workflow. This interprofessional hierarchy impacted the nurses' understanding of their role and profession, as their efforts were not included as a part of the improvement of the patient's understanding of the situation. The focus was on getting the job done and making the patient ready for the “real” physical rehabilitation aiming at improving functioning in accordance with the biomedical discourse.

### Solving of practical tasks vs. inclusion and conversation

The example with the morning routine emphasizes the strong focus and priority on training as the way to ensure rehabilitation progress. This is exemplified by spending the time on ensuring the patient's optimal physical condition for training. This included that the patient was well-rested, pain was managed, food had been consumed, morning routines involving personal hygiene had been completed, and supportive brace devices had been fitted. The mental preparation to make the best of the physical training seemed not to be important or require any specific effort. A change happened when the patient needed help to take a shower and the nurse could not continue her work getting more patients ready at the same time. The nurse then used this short period of time to build a dialogue with the patient, although it was time for emptying bladder and bowels and taking a shower. This showed that the nurse-patient dialogue was central to the nurse—not just to get the task done but to understand the patient and his or her situation. The dialogic space was acknowledged as creating understanding and acknowledgment leading to rehabilitation.

The question is why the opportunity to start a dialogue was seized while solving practical tasks instead of focusing on how the practical tasks could contribute to and promote training? Furthermore, the time-constrained dialogue during practical personal hygiene routines limited the patient's ability to voice his/her perspective and the nurse's ability to adapt her nursing to ensure the best conditions for rehabilitation. The explanation may be that nurses do not have a practice for only entering into a dialogue with the patient. Nursing was focused on practical tasks and the nurses' new recognition of the importance of the space for dialogue required a changed understanding and practice of the profession.

### Physical training vs. understanding of self and the situation to promote recovery

Considering training to improve physical functioning as the most important task revealed a production logic. This biomedical approach may be in line with the value of the professions offering this service. This differed from the nurses' perception as they experienced not getting the space to perform their care. They lacked their preferred biopsychosocial understanding to unfold their nursing care for the patient by focusing on his/her lifeworld. We saw how nurses struggled to legitimize care and conversation by investigating if values such as time for care and time for dialogue could be considered equally important as the production logic by incorporating these elements in nursing to achieve legitimacy in the organization. In the current situation, the biomedical discourse dominated the biopsychosocial approach in the way rehabilitation was prioritized. These elements contrasted with the nurses’ renewed recognition of the importance of listening to the patient's perspective to promote the organization of rehabilitation, increase patient compliance as well as increase meaningfulness of the rehabilitation stays in relation to patients' future life.

Even though the project was successful in supporting the biopsychosocial discourse and approach to rehabilitation among nurses, the implementation faced challenges ascribed to the dominating biomedical discourse and approach. This was found to be a dominating discourse in the healthcare system studied. From the perspective of archaeological knowledge (36), this discourse was prominent in an era and included a “world view” (paradigm), knowledge, understanding, and perspective on disease, diagnosis, and treatment. Foucault focused primarily on psychiatry, but the logic is homologous to existing rehabilitation practices. In each era, knowledge is constructed about disease and treatment. This involves power and forms of subjectivity, which in this context should be understood as the professional identity, function, and role of a nurse. In this way, discourses are understood as more than just ways of thinking and making sense; discourses also involve power and ways to act and organize the prominent discourse (42). The dominating biomedical discourse with a specific focus on solutions and actions as well as a specific understanding of treatment had occupied the way treatment and nursing were organized and performed in the unit. This was reflected in e.g., the prioritization of professional actions and activities based on the dominant biomedical discourse, including an understanding of disease, treatment, care, and rehabilitation.

The co-researchers perceived the other professions such as physiotherapists, occupational therapists, and doctors as opponents. The assumption was that if the other professions acknowledged their legitimacy, things could be changed. However, based on the prominent logic and discourse, it was not within the power of the other professions to provide legitimacy. The original biomedical logic of their professions was the foundation for organizing the highly specialized rehabilitation offers; the organizational system maintained and reproduced its own logic and discourse. It was not in the power of any individual or group to change the intertwined and branched system behind the organization. Metaphorically speaking, even the miller gets milled in the mill as even the professions that installed the biomedical approach are caught within and unable to change it themselves. The system is almost unwavering, which ensures that everybody contributes to the common production with the same unwavering attitude and discourse making the change that nurses find necessary almost impossible. Even though the current study was facilitated by an advisory board and the interdisciplinary management, only a value-based change was seen among the parties involved, i.e., no organized room for dialogue in rehabilitation was created between patients and nurses. The spread of unanimous values in the organization should make it possible for patients to participate and enable them to promote their perspectives and for everybody to adapt their services to optimize the patients' rehabilitation. However, it is reasonable to assume that the organization of the healthcare system must change to enable this local change.

## Discussion

We were surprised that nurses had difficulties in gaining acceptance for taking the time to have dialogues with patients about their situation and in this way legitimize this as a part of their work. In the micro-analysis, prioritizing to **finish on time**, **solve practical tasks**, and **physical training** stood out, all in line with a biomedical approach and discourse. This prioritization contrasted with the nurses' striving to ensure **using all activities related to rehabilitation such as inclusion, conversation,** and **understanding of self and the situation to promote recovery**. This contrast indicated that the focus of rehabilitation was biomedical and that the shift from a biomedical tradition to a biopsychosocial understanding was not reflected and materialized in the overall practice. Moving the analysis to the macro-level showed why the nurses failed; it showed that as a small group in the organization, they were given the opportunity to explore their own practice, and they discovered the discrepancy to their innermost professional beliefs. Not being able to change the conditions of their daily work made the co-researchers feel caught in the painful dilemma of not being true to their beliefs.

The dilemma was not only that they were not able to reach the goal of the nursing profession. The lack of change to a biopsychosocial approach meant that the rehabilitation unit did not work in line with the values and beliefs of the health professionals. The biopsychosocial approach illustrated by the ICF model is the recommended approach in rehabilitation (22). This approach is supposed to replace the biomedical approach which focuses on physical improvement with the broader and more holistic biopsychosocial approach (43). The bodily improvement is still important because the better the bodily damage is restored, return to life as it was may be possible. Thus, if there is a potential for improvement, the need for adaptation to the disability can be reduced. However, in many cases, and almost in all cases of spinal cord injury, the bodily condition and threats from the possible consequences of the injury may be tormenting and challenging to the patient's existential balance (3, 10). This underscores the importance of the biopsychosocial approach and highlights a need for attention to the general health situation. Moreover, personal and environmental factors to individualize the effort are important to increase activities and participation as a result of the improved functioning. Thus, the model aims at identifying the rehabilitation potential and the task in the wider context (21).

A dominant biomedical discourse was suggested to explain why the nurses were challenged when trying to implement the results of the AR project. The dominant biomedical discourse prescribes (subject positions) that the nurses must perform the work based on a specified number of activities within this discourse—understanding care, treatment, and rehabilitation. At the same time, the result of the project rested on a biopsychosocial understanding, which required new activities in the form of time for dialogue and individual adaptation to the individual patient's wishes, needs, and opportunities. Since the system was task-oriented in accordance with the biomedical discourse, there was no free time for practicing the new understanding and activities. The nurses solved this conflict individually and jointly by doing both at the same time i.e., they ran faster to both honor the biomedical requirements and experiment with the new biopsychosocial understanding.

The predominance of the biomedical discourse to be a barrier to patient participation was also found by Joergensen et al. (44). In their study of involving patients' perspectives in a psychiatric context, the biomedical approach was prominent. Also here, nurses struggled with implementing nursing care that involved meeting the patients with openness, encouragement, and trust pointing to organizational structures and the biomedical, and paternalistic framework (44, 45). With this dominating discourse affecting patient participation and the framework for patients' recovery (44, 45), nurses were prevented from performing rehabilitation nursing because this entails consoling, conserving, and integrating functions that simply cannot be carried out without a dialogical approach (23, 24). The discrepancy between the need to work biopsychosocially and the dominating discourse may add to the struggle nurses have to describe their role and function in various rehabilitation settings (46–51).

A frequent reason for few or missing changes is the lack of support from management and involvement of all professionals as well as prioritizing integration of the new practice. However, based on our rereading and reinterpretation of data in our AR study, we identified unrecognized discourses and associated values as barriers to change. Thus, it may be necessary to explore more organizational layers, frameworks, basic assumptions, and organizational structures when conducting a project. In our study, everyone thought that WHO (21) recommendations to use the biopsychosocial approach were followed, but our analysis of the daily practice showed how the biomedical discourse was still predominant. The challenge may be that the barriers may not disclose before the implementation fail, as in our AR study. It was not sufficient that management, other nursing staff, and other professional groups were positive. This fact made us aware that the battle to create space and room for achieving the patient's perspective should not be fought with the other professional groups.

A change in an organization's discourse requires a joint effort. A collaborative team process is necessary as the organizational biomedical framework would also limit the biopsychosocial approach for all health professionals. So, by revealing that the nurses alone cannot create their own isolated space to work in accordance with the biopsychosocial discourse in rehabilitation: The whole organization must collaborate to achieve the WHO goals for rehabilitation (20). This awareness is important to uncover organizational barriers to change. In this case, the biomedical discourse has been extremely beneficial in the organization of health services in relation to saving patients' lives; however, it has not led to the desired goal in rehabilitation units. Thus, there is great value in being critically and constructively aware of the problem, the use of language and actions as well as the lack of actions in relation to changes in the organizational context.

## Conclusion

Our study and analysis show a gap between a stated organizational goal of working predominantly from the biopsychosocial approach and actions which in practice were organized from the biomedical approach. The biomedical approach with its related discourses and actions are so ingrained and rooted in a long institutional tradition that individuals or groups of professionals cannot change it alone. According to our research and analysis, it requires a common recognition, awareness and effort involving the mutually agreed efforts of an entire department, and sometimes the entire organization. The implication of our follow-up discourse analysis is that the dominating discourse must be uncovered followed by a reorganization of discourses if changes are to be implemented and patient participation can occur. This is a difficult task because dominant discourses such as biomedical-oriented health care calls for a larger organizational change involving leaders and all staff groups. By developing a mutual organizational understanding, it may be possible to implement a new practice. In rehabilitation nursing this means that the biopsychosocial approach is needed to allow the patient's agenda to be in focus. It follows from this that the organization must allocate time and resources for dialogue and patient involvement. Furthermore, the organization must work systematically to incorporate the biopsychosocial approach into practice for all professional groups. Thus, staff could follow their professional beliefs and values. This will lead to a better, more coordinated rehabilitation by foregrounding the biopsychosocial approach, and thereby generating greater value and quality in the patient's rehabilitation.

## Data Availability

The paper is theoretical and data are reflections on a larger study and now *per se* available by contacting author Randi Steensgaard. Requests to access these datasets should be directed to Randi Steensgaard rst@specialhospitalet.dk.
